# Supplementary material for: A data science approach for multi-sensor marine observatory data monitoring cold water corals (Paragorgia arborea) in two campaigns
Source: PLoS One. 2023 Jul 19;18(7):e0282723. doi: 10.1371/journal.pone.0282723 (PMC10355400; doi:10.1371/journal.pone.0282723)
Supplement: S1 Fig — The background of image B) is very dark, such that the corals, in particular Cb, cannot be visually assessed well. (PDF) [file pone.0282723.s001.pdf]

S1 Fig: Comparison of gamma-corrected image (A) and original image (B)

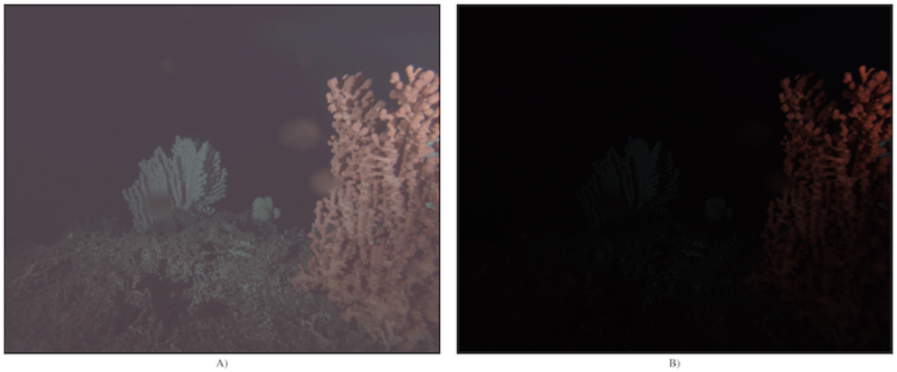

S1 Table: Additional evaluation results for the segmentation models

Validation results and additional performance measures for the segmentation models.

Table 1. Jaccard scores per segmentation model and test or validation dataset.

Model	Measure	Class l	$\mathcal{I}_{1,\text{val}}$	$\mathcal{I}_{1,\text{test}}$	$\mathcal{I}_{1,\text{test}'}$	$\mathcal{I}_{2,\text{val}}$	$\mathcal{I}_{2,\text{test}}$
f_1	$\bar{J}$	-	(0.964)	0.947	0.840	0.638	0.672
f_2	$\bar{J}$	-	0.876	0.880	0.953	(0.937)	0.932
f_*	$\bar{J}$	-	(0.966)	0.925	0.961	(0.939)	0.939
f_1	J_l	$L(C_r)$	(0.968)	0.962	0.951	0.886	0.886
f_2	J_l	$L(C_r)$	0.954	0.949	0.956	(0.935)	0.941
f_*	J_l	$L(C_r)$	(0.966)	0.964	0.960	(0.935)	0.943
f_1	J_l	$L(C_b)$	(0.938)	0.896	0.612	0.072	0.175
f_2	J_l	$L(C_b)$	0.702	0.718	0.922	(0.893)	0.870
f_*	J_l	$L(C_b)$	(0.945)	0.828	0.939	(0.900)	0.888

Values in brackets refer to datasets used for optimizing the respective model. If a performance measure is specific to a class label l, l is given in the column "Class l" (see also Eq (1) in the main document).

Table 2. Recall, Precision, and F_1 scores per segmentation model and test or validation dataset.

Model	Measure	Class l	$\mathcal{I}_{1,\text{val}}$	$\mathcal{I}_{1,\text{test}}$	$\mathcal{I}_{1,\text{test}'}$	$\mathcal{I}_{2,\text{val}}$	$\mathcal{I}_{2,\text{test}}$
f_1	$\bar{P}$	-	(0.979)	0.973	0.967	0.937	0.908
f_2	$\bar{P}$	-	0.913	0.935	0.969	(0.964)	0.970
f_*	$\bar{P}$	-	(0.985)	0.979	0.978	(0.967)	0.974
f_1	P_l	$L(C_r)$	(0.979)	0.974	0.957	0.900	0.900
f_2	P_l	$L(C_r)$	0.981	0.979	0.967	(0.959)	0.966
f_*	P_l	$L(C_r)$	(0.987)	0.983	0.972	(0.960)	0.970
f_1	P_l	$L(C_b)$	(0.962)	0.949	0.971	0.932	0.845
f_2	P_l	$L(C_b)$	0.772	0.840	0.945	(0.940)	0.952
f_*	P_l	$L(C_b)$	(0.977)	0.963	0.969	(0.948)	0.960
f_1	$\bar{R}$	-	(0.985)	0.973	0.867	0.677	0.713
f_2	$\bar{R}$	-	0.947	0.929	0.982	(0.970)	0.958
f_*	$\bar{R}$	-	(0.980)	0.943	0.982	(0.970)	0.962
f_1	R_l	$L(C_r)$	(0.989)	0.987	0.994	0.983	0.983
f_2	R_l	$L(C_r)$	0.971	0.969	0.988	(0.974)	0.974
f_*	R_l	$L(C_r)$	(0.979)	0.980	0.988	(0.973)	0.972
f_1	R_l	$L(C_b)$	(0.974)	0.941	0.623	0.073	0.181
f_2	R_l	$L(C_b)$	0.886	0.831	0.974	(0.947)	0.909
f_*	R_l	$L(C_b)$	(0.967)	0.854	0.968	(0.947)	0.922
f_1	$\bar{F_1}$	-	(0.982)	0.973	0.904	0.684	0.738
f_2	$\bar{F_1}$	-	0.929	0.932	0.976	(0.967)	0.964
f_*	$\bar{F_1}$	-	(0.983)	0.960	0.980	(0.968)	0.968
f_1	$F_{1,l}$	$L(C_r)$	(0.984)	0.980	0.975	0.940	0.940
f_2	$F_{1,l}$	$L(C_r)$	0.976	0.974	0.978	(0.966)	0.970
f_*	$F_{1,l}$	$L(C_r)$	(0.983)	0.982	0.980	(0.966)	0.971
f_1	$F_{1,l}$	$L(C_b)$	(0.968)	0.945	0.759	0.135	0.298
f_2	$F_{1,l}$	$L(C_b)$	0.825	0.836	0.959	(0.944)	0.930
f_*	$F_{1,l}$	$L(C_b)$	(0.972)	0.906	0.968	(0.947)	0.941

Values in brackets refer to validation datasets used for optimizing the respective model. If the applied performance measure is specific to one class label l, l is given in the column "Class l" (see also Eq (1) in the main document).

S1 Text: Hyperparameter selection for segmentation and classification models

Hyperparameter optimization was done analogously for the segmentation and classification models. First, we tested a set of hyperparameters using four-fold cross validation using the training data. Second, using the parameters of the model achieving the highest macro F_1 score in the first step, we tested combinations of various data augmentation operations. We used four-fold cross validation using the training images in this step as well.

After all cross validation steps were finished, the best combination of hyperparameters and augmentation methods was used to train a model using all images in the training dataset. The U-Nets were trained for 200 epochs, while each ResNet was trained for 100 epochs. After each epoch, a model evaluation was conducted with respect to the macro-averaged F_1 score $\bar{F}_1$. The weights with the best $\bar{F}_1$ were kept for model testing and application. As a loss function, the cross entropy loss was used.

U-Net parameters

For the U-Net models f_1 and f_2, own cross validation experiments were conducted. For f_*, only the best hyperparameter and augmentation configurations for f_1 and f_2 were compared.

For the U-Net models, normalization of the images was done using mean and standard deviation calculated from the training images. All U-Net models were trained from random initialization. In our U-Net architectures, convolutional layers in the upsampling and downsampling paths are always arranged in pairs, except for the final layer generating the segmentation masks. This is also the case in the original architecture proposed by Ronneberger et al. [1]. We define the depth of a U-Net architecture as the number of pairs of convolutional layers located after the upsampling operations in the upsampling path, which equals the number of skip connections. For more information on the U-Net architecture, please refer to Ronneberger et al. [1].

We use two U-Net architectures, which differ from the original architecture proposed by Ronnerberger et al.: The first (UNet7) has a depth of 7, with each convolutional layer in the network except for the final 1-channel segmentation layer having 64 output feature maps. The second architecture (UNet4) is very similar to the UNet7 architecture, but has a depth of 4.

The only augmentation method used in training of the final U-Net models is the "Shift" augmentation method, which applies random shifts by at maximum 20% of the image width and height, respectively, to the images and the segmentation masks. Edges were padded using reflected pixels. Gamma correction with $\gamma = 0.3$ was applied during image preprocessing in order to increase brightness and contrast. An overview of the selected hyperparameters for each segmentation model can be seen Table 1.

Table 1. Hyperparameters selected for the segmentation models.

Hyperparameter	f_1	f_2	f_*
Augmentation	Shift	None	None
Batch size	1	1	1
gamma	0.3	0.3	0.3
model type	UNet7	UNet 4	UNet4
Normalization	yes	yes	yes
Optimizer	Adam[2]	Adam	Adam
Learning rate (LR)	0.0001	0.0001	0.0001
LR Scheduling	None	None	None

ResNet parameters

Optimization of models trained on data from both time periods Γ_1 and Γ_2 was done analogously to U-Net model optimization for f_* (see above).

Augmentation methods applied while training classification models include shifts of up to 20% of the image width and height (see above). Furthermore, the rotation augmentation method (Rot) was used. Using this method, the input images are rotated by a randomly selected angle. Possible values are 0°, 90°, 180°, or 270°, which are selected with equal probability.

Further augmentation methods applied include shearing augmentation (Shear) with a randomly selected shearing angle between -20 and 20 degrees. Here, the result images are padded with zeros. If used during training, horizontal flips (HFlip) and vertical flips (VFlip) are applied with a chance of 50%.

The mean and standard deviation used for normalization were taken from the PyTorch default image preprocessing parameters for neural networks initalized with weights pretrained on the ImageNet dataset. The used mean is $\mu = [0.485, 0.456, 0.406]$ and the standard deviation is $\sigma = [0.229, 0.224, 0.225]$ (See PyTorch ResNet50 documentation: https://pytorch.org/vision/stable/models/generated/torchvision.models.resnet50.html#torchvision.models.ResNet50_Weights [accessed at 4 August 2022]).

It can be seen that many optimized hyperparameters are similar or the same for models for both periods Γ_1 and Γ_2 as well as corals C_r and C_b. An overview of the selected hyperparameters for each classification model can be seen in Table 2.

Table 2. Hyperparameters selected for the classification models.

Hyperparameter	$g_{1,r}$	$g_{2,r}$	$g_{*,r}$	$g_{1,b}$	$g_{2,b}$	$g_{*,b}$
Augmentation	VFlip	Rot<br>Shear<br>Shift	Rot<br>Shear<br>Shift	Rot<br>HFlip<br>VFlip	None	None
Batch size	64	64	64	64	32	32
Imagenet weights	Yes	Yes	Yes	Yes	Yes	Yes
Normalization	Yes	Yes	Yes	Yes	Yes	Yes
Optimizer	Adam	Adam	Adam	Adam	Adam	Adam
Learning rate (LR)	0.0001	0.0001	0.0001	0.0001	0.0001	0.0001
LR scheduling	None	None	None	None	None	None

References

[1] Ronneberger O, Fischer P, Brox T. U-Net: Convolutional Networks for Biomedical Image Segmentation. In: Medical Image Computing and Computer-Assisted Intervention (MICCAI). vol. 9351 of LNCS. Springer; 2015. p. 234–241. Available from: `http://lmb.informatik.uni-freiburg.de/Publications/2015/RFB15a`.

[2] Kingma DP, Ba J. Adam: A Method for Stochastic Optimization. In: Bengio Y, LeCun Y, editors. 3rd International Conference on Learning Representations, ICLR 2015, San Diego, CA, USA, May 7-9, 2015, Conference Track Proceedings; 2015.Available from: `http://arxiv.org/abs/1412.6980`.

S2 Fig: Plots of Input Feature Sensor Data

The polyp activity time series generated from the stereo camera images for the red *Paragorgia* C_r is shown together with time series of depth, temperature, and the three current velocity components v_1, v_2, and v_3. For each hour, a median value of the current data is shown. Gaps in the data are visible in the plots. Smoothing of the polyp activity data was applied as described in S5 Text using Gaussian smoothing with $\sigma = 10$.

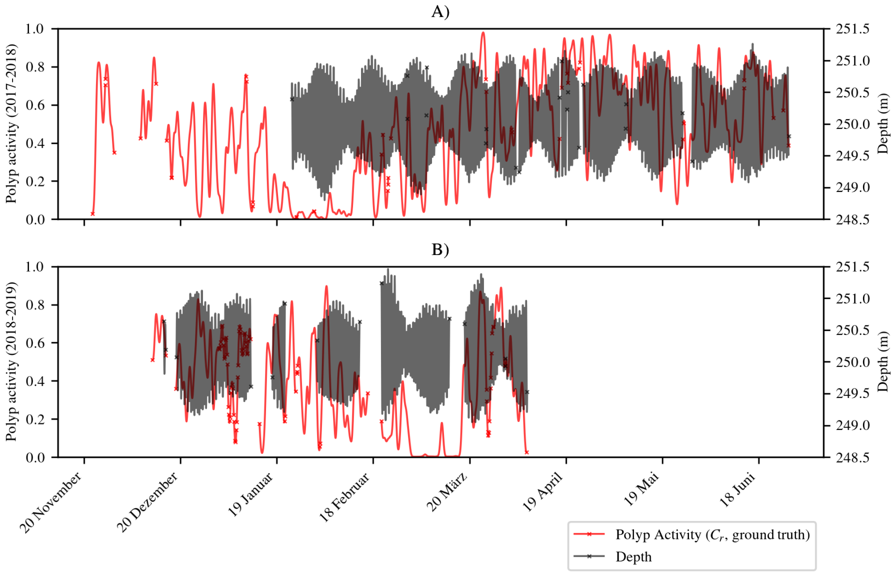

Fig 1. Polyp activity of the red *Paragorgia* plotted together with the depth data.

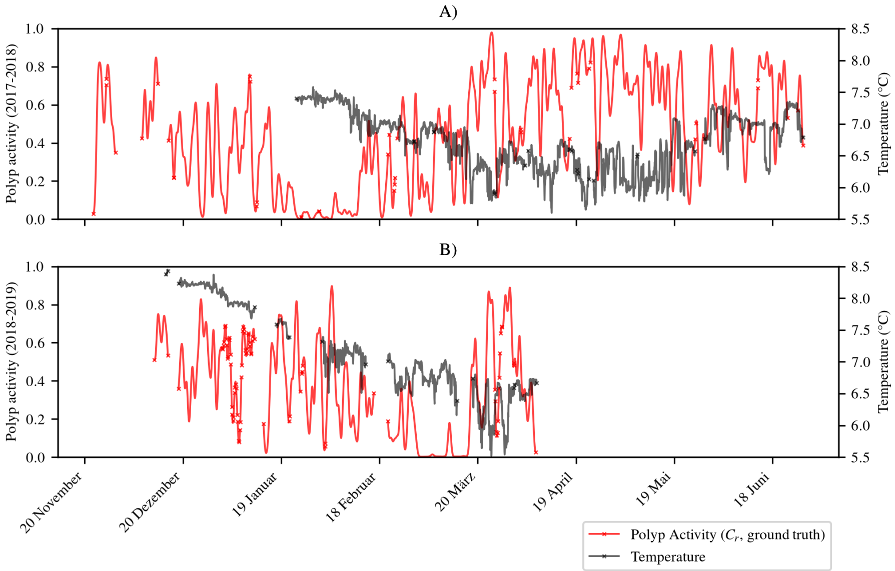

Fig 2. Polyp activity of the red *Paragorgia* plotted together with the temperature data.

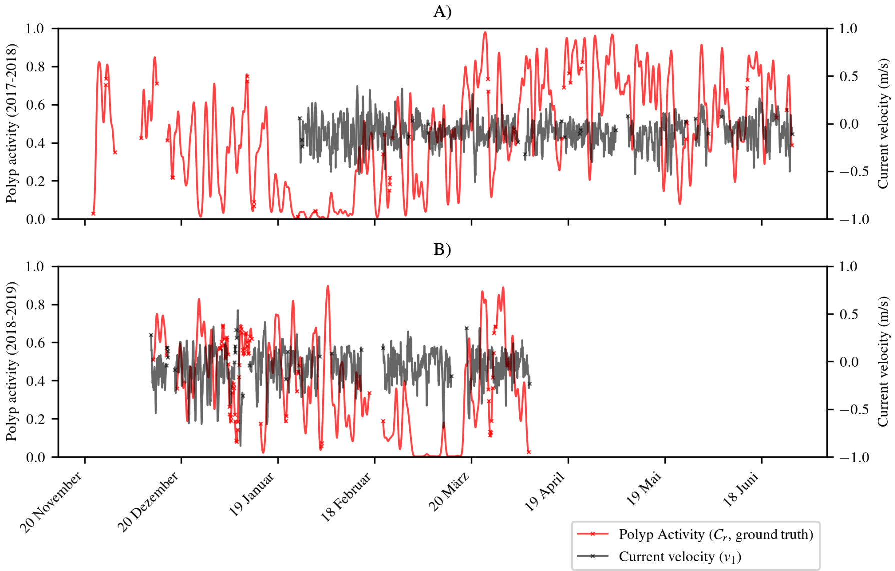

Fig 3. Polyp activity of the red *Paragorgia* plotted together with the current velocity component v_1 data.

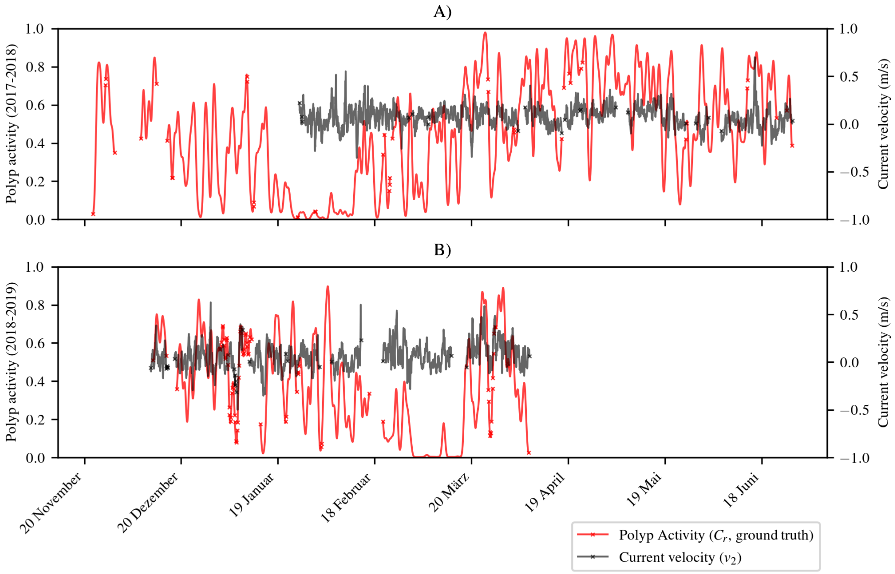

Fig 4. Polyp activity of the red *Paragorgia* plotted together with the current velocity component v_2 data.

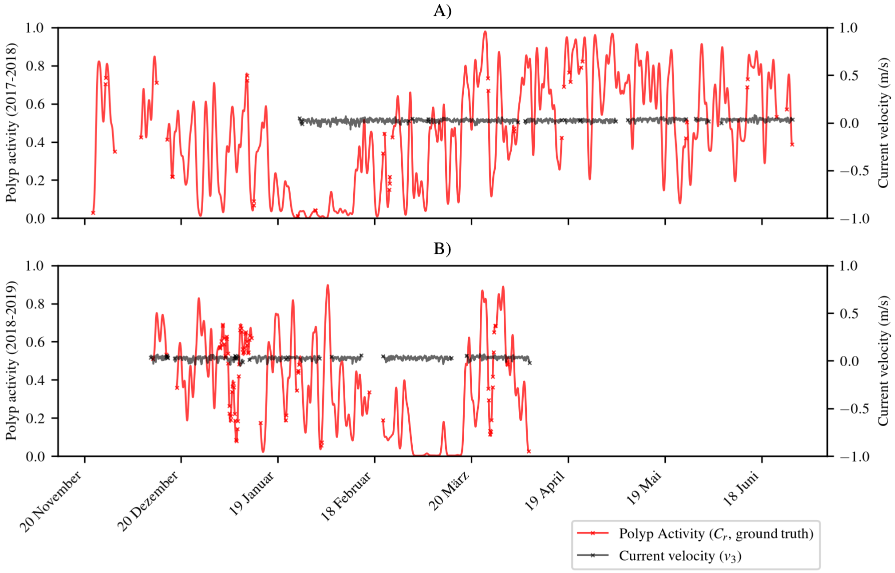

Fig 5. Polyp activity of the red *Paragorgia* plotted together with the current velocity component v_3 data.

S2 Table: Test and validation macro-averaged F_1 scores ($\bar{F_1}$) of the classification models.

Model	Coral	$\mathcal{D}^c_{1,\text{val}}$	$\mathcal{D}^c_{1,\text{test}}$	$\mathcal{D}^c_{1,\text{test}'}$	$\mathcal{D}^c_{2,\text{val}}$	$\mathcal{D}^c_{2,\text{test}}$
$g_{1,r}$	C_r	(0.973)	0.963	0.979	0.962	0.936
$g_{1,b}$	C_b	(1.000)	0.957	0.944	0.888	0.895
$g_{2,r}$	C_r	0.972	0.952	0.990	(0.983)	0.963
$g_{2,b}$	C_b	0.948	0.905	0.923	(0.957)	0.952
$g_{*,r}$	C_r	(0.975)	0.968	0.990	(0.982)	0.960
$g_{*,b}$	C_b	(0.986)	0.957	0.929	(0.973)	0.951

Values in brackets indicate that the dataset was used for optimizing the tested model. Note that cross-time period validation sets can be seen as additional test sets as these sets were not used for optimizing the respective models.

S2 Text: Patch generation for polyp activity classification

Our method for patch generation can be applied using manually annotated masks or masks generated e.g. by a CNN. As the masks generated by the segmentation models are smaller than the original images, they are upscaled before patch generation. For a given coral c, patches $D^i_{j=0,\ldots,n_i}$ from an image I_i are generated as follows: An image patch is defined as

$$D^i_j = \{I_i(x,y) | x_0(D^i_j) \leq x < x_0(D^i_j) + 128, \quad (1)$$

$$y_0(D^i_j) \leq y < y_0(D^i_j) + 128\} \quad (2)$$

and can be written as $D^i_j = (I_i, x_0, y_0)$, where (x_0, y_0) define the top left corner of D^i_j in I_i. Each image patch has a corresponding mask patch $Q^i_j = (M_i, x_0, y_0)$, which is defined accordingly. Each patch D^i_j is assigned a weight $w^i_j \in [0,1]$ according to the share of pixels labeled as c in the corresponding mask patch Q^i_j.

Positions for patch extraction are defined as follows: First, a rectangular patch extraction region B_i is defined in image I_i. B_i is defined as a box around the center of the bounding box of the region segmented as coral c $O_i = \{(x,y) | M_i(x,y) = L(c)\}$ such that all pixels in O_i lie within B_i. The edge lengths of B_i are multiples of 128. If B_i overlaps with an edge of I_i, it is shifted such that is completely located within I_i.

Prior to extracting the patches, the background in the image is masked in order to remove unnecessary information. The masked image I'_i is defined as follows:

$$I'_i(x,y) = \begin{cases} I_i(x,y), & (x,y) \in O_i \\ (0,0,0), & \text{else.} \end{cases} \quad (3)$$

The content of O_i in I'_i is subdivided into square patches $D^i_{j=0,\ldots,n_i}$ without an overlap of the extracted patches.

S3 Text: Polyp activity estimation for the "blue" coral C_b

The coral C_b is only visible in images recorded from camera viewing angles θ_1 and θ_2 during Γ_1' and Γ_2. In images recorded by the optical stereo camera sensor K_0 from viewing angle θ_1, the visible part of C_b is small, while K_1 recorded the entire coral C_b from this viewing angle. In addition, many images recorded during Γ_1 do not show C_b, but for these images, no information on the camera position is given, i.e the images would need to be viewed to see if they show coral C_b and thus cannot be automatically excluded from the analysis.

To reduce the risk of falsely segmenting C_b in images where in fact it is not present, we exclude all images for which less than 1000 C_b pixels were segmented before activity estimation. Images with more than 30000 C_b pixels segmented are also excluded as they are considered outliers. Sizes are computed based on the segmentation masks with a size of 551×688. In addition, we only analyze θ_2 images from stereo camera sensor K_0 and θ_1 and θ_2 images from stereo camera sensor K_1 with respect to C_b for period Γ_2. To differentiate between camera angles is not necessary for Γ_1' as the smaller regions are already excluded using the lower threshold of 1000 pixels. For C_r, we use all available images captured before 7 April 2019 and do not apply any thresholds.

S4 Text: Evaluation metrics and accuracy assessment

The segmentation models are evaluated using the Jaccard score J [1], which can be defined for a class l (i.e. for the binary case) as

$$J_l = \frac{TP_l}{TP_l + FP_l + FN_l}, \tag{1}$$

where TP_l, FP_l, and FN_l are true positive, false positive, and false negative classifications , here pixel classifications with respect to class l [2, 3].

For evaluation of the classification models, we use the F_1-score (also called F-measure), which is based on the precision (P) and recall (R) measures. Their definition with respect to a given class l are as follows [3, 4, 5, 6]:

$$P_l = \frac{TP_l}{TP_l + FP_l} \tag{2}$$

$$R_l = \frac{TP_l}{TP_l + FN_l}, \tag{3}$$

$$F_{1,l} = \frac{2P_l R_l}{P_l + R_l} = \frac{2TP_l}{2TP_l + FP_l + FN_l}, \tag{4}$$

To assess the overall performance of a segmentation or classification model, we use the macro-average of the performance measures introduced above. The macro-averaged performance measure is an unweighted average of the performance measures for each class. For a performance measure E, the macro average will be denoted as $\bar{E}$ and is defined as follows [3, 7, 8]:

$$\bar{E} = \frac{1}{N} \sum_{l=1}^{N} E_l, \tag{5}$$

where N is the number of classes available.

References

[1] Jaccard P. The Distribution of the Flora in the Alpine Zone. The New Phytologist. 1912;11(2):37–50.

[2] Taha AA, Hanbury A. Metrics for evaluating 3D medical image segmentation: analysis, selection, and tool. BMC Medical Imaging. 2015;15:29. doi:https://doi.org/10.1186/s12880-015-0068-x.

[3] Lipton ZC, Elkan C, Naryanaswamy B. Optimal Thresholding of Classifiers to Maximize F1 Measure. In: Calders T, Esposito F, Hüllermeier E, Meo R, editors. Machine Learning and Knowledge Discovery in Databases. Berlin, Heidelberg: Springer Berlin Heidelberg; 2014. p. 225–239.

[4] Fawcett T. Introduction to ROC analysis. Pattern Recognition Letters. 2006;27:861–874. doi:10.1016/j.patrec.2005.10.010.

[5] Sokolova M, Lapalme G. A systematic analysis of performance measures for classification tasks. Information Processing & Management. 2009;45:427–437. doi:10.1016/j.ipm.2009.03.002.

[6] Ferri C, Hernández-Orallo J, Modroiu R. An experimental comparison of performance measures for classification. Pattern Recognit Lett. 2009;30:27–38.

[7] Tsoumakas G, Katakis I, Vlahavas I. Random k-Labelsets for Multilabel Classification. IEEE Transactions on Knowledge and Data Engineering. 2011;23(7):1079–1089. doi:10.1109/TKDE.2010.164.

[8] Yang Y. An Evaluation of Statistical Approaches to Text Categorization. Inf Retr. 1999;1(1–2):69–90. doi:10.1023/A:1009982220290.

S5 Text: Hyperparameter and feature selection for LSTM

First, we tested several parameter combinations for LSTM. Second, we tested all combinations of the input features depth, temperature, and the three current components v_1, v_2, and v_3. Third, we tested all combinations of the best ten parameter configurations and the best ten feature combinations. The parameter and feature combination achieving the lowest mean absolute error (MAE) predicting $\hat{a}(t)$ for the test time period from 22 March 2018, 15:00 until 12 April 2018, 14:00 was used to generate the experiment results described in Section 4.5.

Our approach for polyp activity prediction using LSTM models (sliding window) is explained in Section 3.6. As a loss function, the mean absolute error is used. For a list of all optimized parameters see Table 1.
The LSTM model architecture finally used consists of two LSTM layers in a row, each layer having an eight-dimensional hidden state. The last layer is connected to a single neuron that outputs the predicted activity value.

Table 1. Selected parameters for the LSTM models.

Parameter	Value
Optimizer	Stochastic gradient descent (SGD) with momentum=0.9
Learning rate	0.01
Subseries length η	6
Training Epochs per model $h_{t'}$	100
Hidden state dimensions	8
LSTM layers	2
Batch size	32

For further information on the SGD optimizer and momentum, please refer to [1].

References

[1] Sutskever I, Martens J, Dahl G, Hinton G. On the importance of initialization and momentum in deep learning. In: Dasgupta S, McAllester D, editors. Proceedings of the 30th International Conference on Machine Learning. vol. 28 of Proceedings of Machine Learning Research. Atlanta, Georgia, USA: PMLR; 2013. p. 1139–1147. Available from: `https://proceedings.mlr.press/v28/sutskever13.html`.

S6 Text: Preprocessing of sensor data in LSTM training and prediction

The LSTM architecture we use has a fixed input vector size. Therefore, measurements for all input features have to be available for all time points in an input subseries S_t for predicting polyp activity $\hat{a}(t)$. If a feature is missing at time point t, the full input vector $s(t)$ is removed, leaving a gap in the time series of input features. Standardization of the input features in $s(t)$ is done after removing values for time points t for which any feature is missing.

If a gap in time series $s(t)$ is smaller than 8 consecutive hours, the sensor data from the η previous, not consecutive, time steps available are used for predicting the polyp activity $\hat{a}(t)$ instead. If the gap is larger than 8 hours, no activity $\hat{a}(t)$ is predicted. The next t to predict $\hat{a}(t)$ for is selected such that η input vectors that are either consecutive or divided by a gap not larger than 8 hours are available as LSTM input.

S7 Text: Data smoothing for visualization

The smoothed time series plots shown in this paper (Fig 7, Fig 10, Fig 1 - Fig 5 in S2 Fig, Fig 1 in S6 Text) are generated as follows: Missing values are interpolated using linear interpolation if data for up to five hours are missing. If the gap is larger, the mean value of the time series is inserted in the full gap. The interpolated time series is smoothed using a Gaussian filter. After smoothing, all values inserted in the interpolation step are removed.

S8 Text: LSTM prediction results and correlations for the blue *Paragorgia*

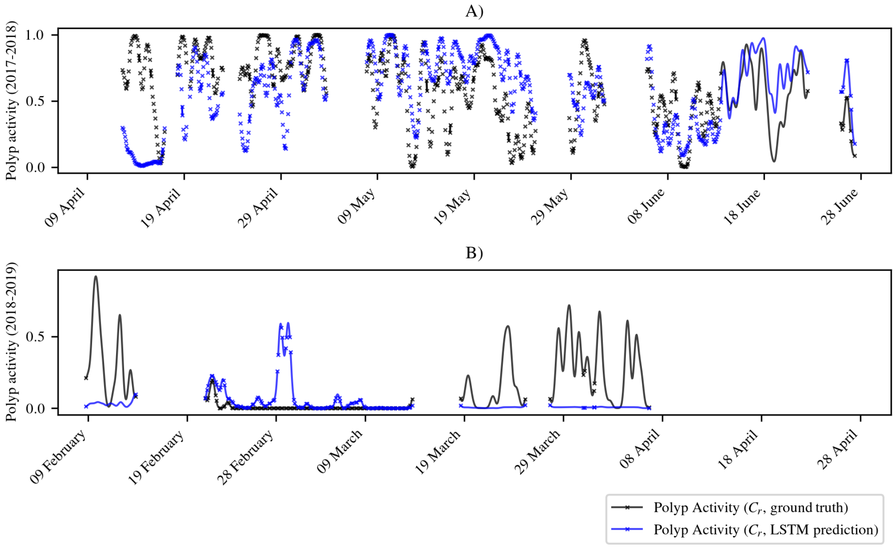

Fig 1. Smoothed result of polyp activity prediction for coral C_b, using our LSTM approach. Plots A) and B) show the results and ground truth for datasets $\mathcal{A}'_1$ and $\mathcal{A}'_2$, respectively. Smoothing was applied as described in S5 Text using a Gaussian filter with $\sigma = 5$. An "x" marker indicates the end of a series of consecutive hourly data points and thus a gap in the data.

$\mathcal{A}'_\tau$ refers to C_b polyp activity analogous to $\mathcal{A}_\tau$ for C_r polyp activity. The MAE for the "blue" colony C_b and $\mathcal{A}'_1$ is 0.344 ($r_S = 0.332,\ p = 1.2 \cdot 10^{-22}$). The MAE for C_b and $\mathcal{A}'_2$ is 0.166 ($r_S = 0.236,\ p = 8.7 \cdot 10^{-14}$). Correlations of "blue" coral activity with the feature time series can be found in Table 1. Correlations are calculated analogous to those for coral C_r (see Section 4.5). A plot of ground truth and predicted polyp activity of C_b can be found in Fig 1. For C_b activity prediction, the same set of input features was used as for C_r activity prediction.

Table 1. Spearman rank correlations and p-values.

	$\mathcal{A}_1'$ ($N = 826$)		$\mathcal{A}_2'$ ($N = 970$)	
Datatype	r_S	p	r_S	p
Depth	-0.046	0.184	-0.013	0.690
Temperature	-0.282	$1.4 \cdot 10^{-16}$	-0.046	0.148
Current velocity v_1	-0.170	$8.5 \cdot 10^{-7}$	-0.070	0.029
Current velocity v_2	0.190	$3.9 \cdot 10^{-8}$	0.158	$7.2 \cdot 10^{-7}$
Current velocity v_3	-0.277	$5.3 \cdot 10^{-16}$	0.016	0.624
Predicted activity	0.332	$1.2 \cdot 10^{-22}$	0.236	$8.7 \cdot 10^{-14}$

The table shows the Spearman rank correlations r_S between the ground truth polyp activity values for the blue *Paragorgia* C_b in the datasets $\mathcal{A}_1'$ and $\mathcal{A}_2'$ and the features used as input for polyp activity prediction using LSTM. Additionally, r_S between C_b ground truth polyp activity in $\mathcal{A}_1'$ and $\mathcal{A}_2'$ and the polyp activities predicted using our LSTM approach is shown. Accompanying p-values are shown for each correlation.
