# Supplementary material for: A data science approach for multi-sensor marine observatory data monitoring cold water corals (Paragorgia arborea) in two campaigns
Source: PLoS One. 2023 Jul 19;18(7):e0282723. doi: 10.1371/journal.pone.0282723 (PMC10355400; doi:10.1371/journal.pone.0282723)
Supplement: S2 Fig — The polyp activity time series a(t) is shown together with the non-image sensor data used as LSTM input features (see Section 3.6). (PDF) [file pone.0282723.s002.pdf]

S2 Fig: Plots of Input Feature Sensor Data

The polyp activity time series generated from the stereo camera images for the red *Paragorgia* C_r is shown together with time series of depth, temperature, and the three current velocity components v_1, v_2, and v_3. For each hour, a median value of the current data is shown. Gaps in the data are visible in the plots. Smoothing of the polyp activity data was applied as described in S5 Text using Gaussian smoothing with $\sigma = 10$.

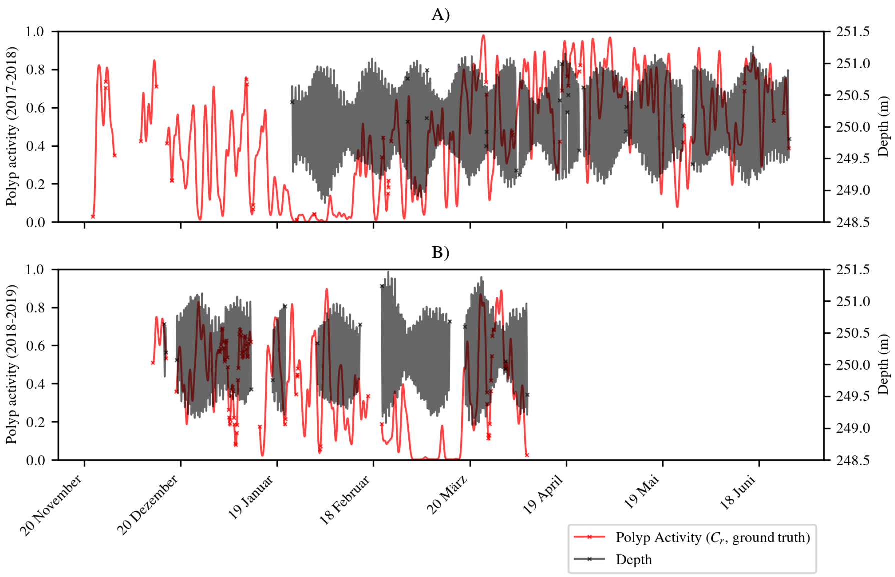

Fig 1. Polyp activity of the red *Paragorgia* plotted together with the depth data.

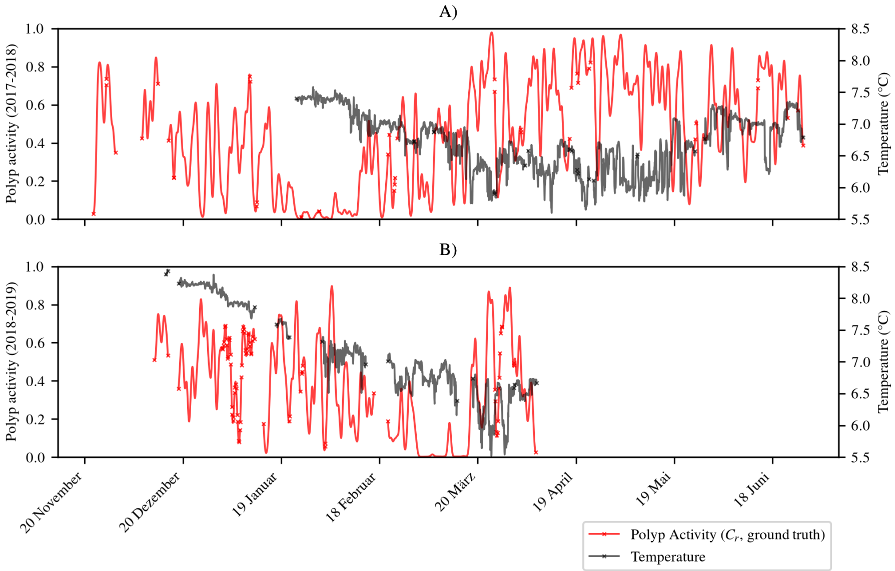

Fig 2. Polyp activity of the red *Paragorgia* plotted together with the temperature data.

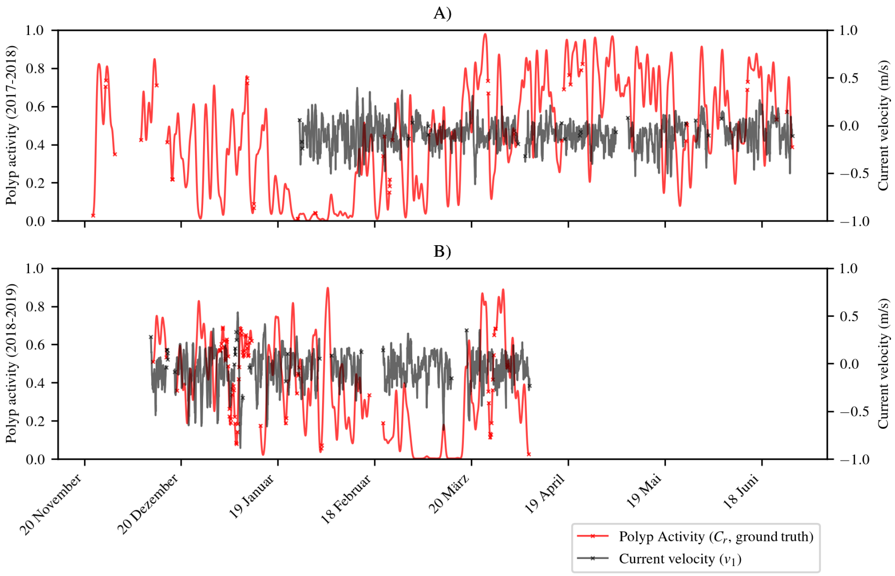

Fig 3. Polyp activity of the red *Paragorgia* plotted together with the current velocity component v_1 data.

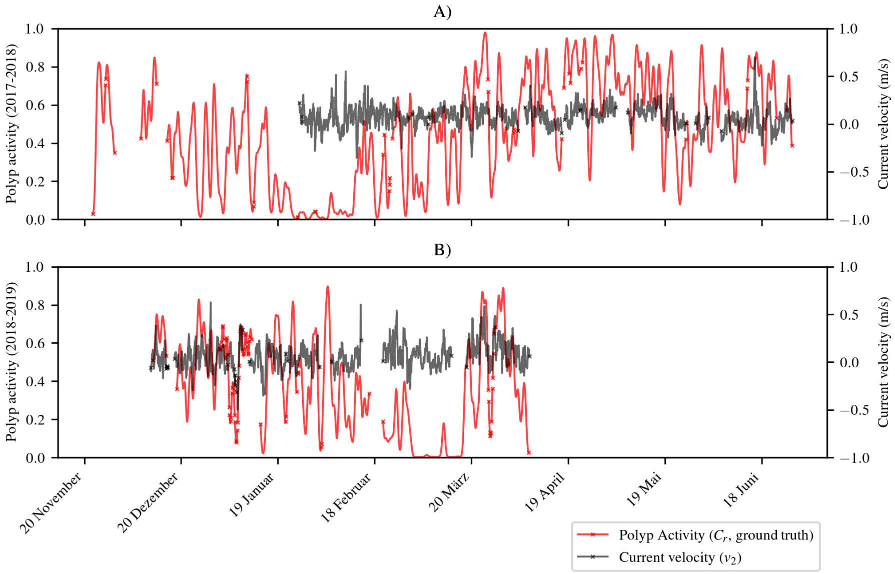

Fig 4. Polyp activity of the red *Paragorgia* plotted together with the current velocity component v_2 data.

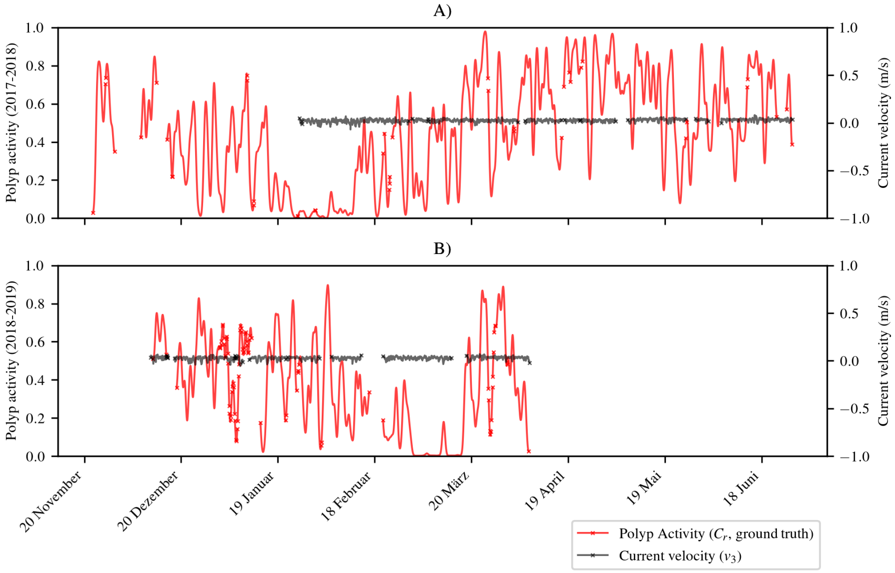

Fig 5. Polyp activity of the red *Paragorgia* plotted together with the current velocity component v_3 data.
