# Supplementary figures and images for: A data science approach for multi-sensor marine observatory data monitoring cold water corals (Paragorgia arborea) in two campaigns
Source: PLoS One. 2023 Jul 19;18(7):e0282723. doi: 10.1371/journal.pone.0282723 (PMC10355400; doi:10.1371/journal.pone.0282723)

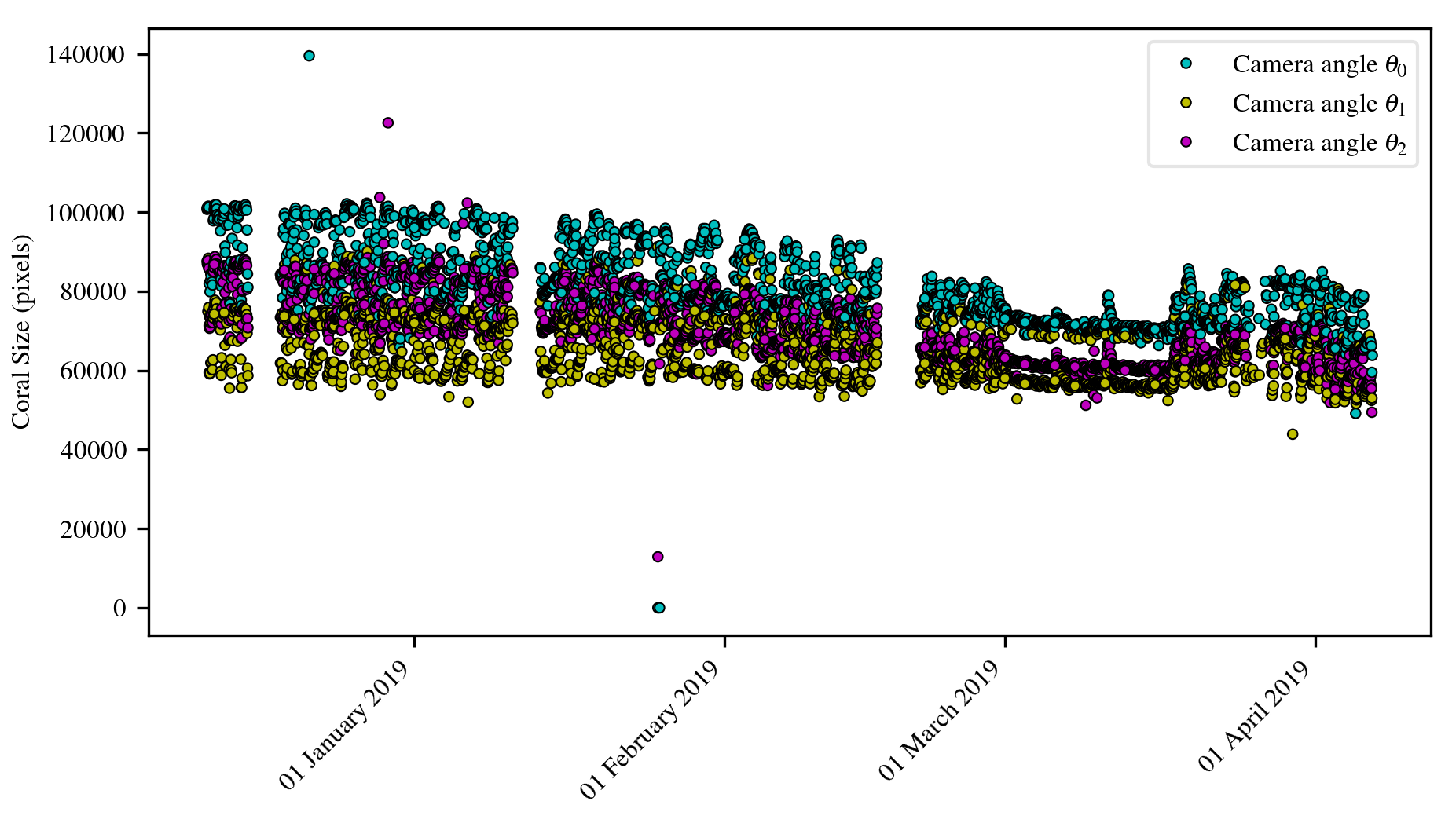

Supplement: S3 Fig — Size of the segmented Cr region in the images recorded by stereo camera sensor K0 during time period Γ2 plotted against time. Regions were segmented by U-Net f2. The sizes were plotted separately according to the camera angle of the corresponding images. (PNG) [file pone.0282723.s003.png]

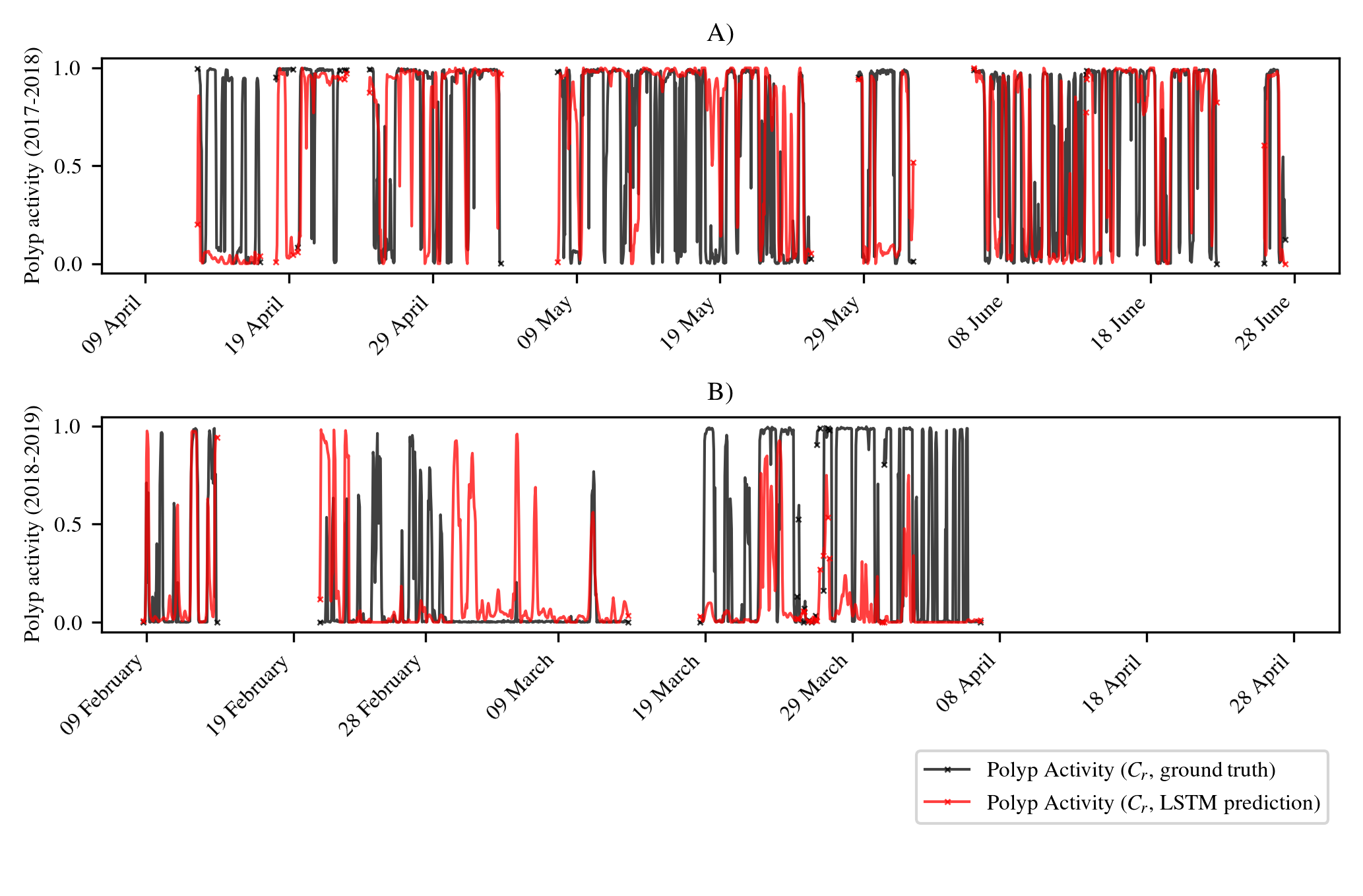

Supplement: S4 Fig — Plot of unsmoothed ground truth and LSTM-predicted Cr activity time series. (PNG) [file pone.0282723.s004.png]
