# Supplementary material for: A data science approach for multi-sensor marine observatory data monitoring cold water corals (Paragorgia arborea) in two campaigns
Source: PLoS One. 2023 Jul 19;18(7):e0282723. doi: 10.1371/journal.pone.0282723 (PMC10355400; doi:10.1371/journal.pone.0282723)
Supplement: S1 Text — A description of the parameter selection scheme used for the segmentation and classification models. (PDF) [file pone.0282723.s005.pdf]

S1 Text: Hyperparameter selection for segmentation and classification models

Hyperparameter optimization was done analogously for the segmentation and classification models. First, we tested a set of hyperparameters using four-fold cross validation using the training data. Second, using the parameters of the model achieving the highest macro F_1 score in the first step, we tested combinations of various data augmentation operations. We used four-fold cross validation using the training images in this step as well.

After all cross validation steps were finished, the best combination of hyperparameters and augmentation methods was used to train a model using all images in the training dataset. The U-Nets were trained for 200 epochs, while each ResNet was trained for 100 epochs. After each epoch, a model evaluation was conducted with respect to the macro-averaged F_1 score $\bar{F}_1$. The weights with the best $\bar{F}_1$ were kept for model testing and application. As a loss function, the cross entropy loss was used.

U-Net parameters

For the U-Net models f_1 and f_2, own cross validation experiments were conducted. For f_*, only the best hyperparameter and augmentation configurations for f_1 and f_2 were compared.

For the U-Net models, normalization of the images was done using mean and standard deviation calculated from the training images. All U-Net models were trained from random initialization. In our U-Net architectures, convolutional layers in the upsampling and downsampling paths are always arranged in pairs, except for the final layer generating the segmentation masks. This is also the case in the original architecture proposed by Ronneberger et al. [1]. We define the depth of a U-Net architecture as the number of pairs of convolutional layers located after the upsampling operations in the upsampling path, which equals the number of skip connections. For more information on the U-Net architecture, please refer to Ronneberger et al. [1].

We use two U-Net architectures, which differ from the original architecture proposed by Ronnerberger et al.: The first (UNet7) has a depth of 7, with each convolutional layer in the network except for the final 1-channel segmentation layer having 64 output feature maps. The second architecture (UNet4) is very similar to the UNet7 architecture, but has a depth of 4.

The only augmentation method used in training of the final U-Net models is the "Shift" augmentation method, which applies random shifts by at maximum 20% of the image width and height, respectively, to the images and the segmentation masks. Edges were padded using reflected pixels. Gamma correction with $\gamma = 0.3$ was applied during image preprocessing in order to increase brightness and contrast. An overview of the selected hyperparameters for each segmentation model can be seen Table 1.

Table 1. Hyperparameters selected for the segmentation models.

Hyperparameter	f_1	f_2	f_*
Augmentation	Shift	None	None
Batch size	1	1	1
gamma	0.3	0.3	0.3
model type	UNet7	UNet 4	UNet4
Normalization	yes	yes	yes
Optimizer	Adam[2]	Adam	Adam
Learning rate (LR)	0.0001	0.0001	0.0001
LR Scheduling	None	None	None

ResNet parameters

Optimization of models trained on data from both time periods Γ_1 and Γ_2 was done analogously to U-Net model optimization for f_* (see above).

Augmentation methods applied while training classification models include shifts of up to 20% of the image width and height (see above). Furthermore, the rotation augmentation method (Rot) was used. Using this method, the input images are rotated by a randomly selected angle. Possible values are 0°, 90°, 180°, or 270°, which are selected with equal probability.

Further augmentation methods applied include shearing augmentation (Shear) with a randomly selected shearing angle between -20 and 20 degrees. Here, the result images are padded with zeros. If used during training, horizontal flips (HFlip) and vertical flips (VFlip) are applied with a chance of 50%.

The mean and standard deviation used for normalization were taken from the PyTorch default image preprocessing parameters for neural networks initalized with weights pretrained on the ImageNet dataset. The used mean is $\mu = [0.485, 0.456, 0.406]$ and the standard deviation is $\sigma = [0.229, 0.224, 0.225]$ (See PyTorch ResNet50 documentation: https://pytorch.org/vision/stable/models/generated/torchvision.models.resnet50.html#torchvision.models.ResNet50_Weights [accessed at 4 August 2022]).

It can be seen that many optimized hyperparameters are similar or the same for models for both periods Γ_1 and Γ_2 as well as corals C_r and C_b. An overview of the selected hyperparameters for each classification model can be seen in Table 2.

Table 2. Hyperparameters selected for the classification models.

Hyperparameter	$g_{1,r}$	$g_{2,r}$	$g_{*,r}$	$g_{1,b}$	$g_{2,b}$	$g_{*,b}$
Augmentation	VFlip	Rot<br>Shear<br>Shift	Rot<br>Shear<br>Shift	Rot<br>HFlip<br>VFlip	None	None
Batch size	64	64	64	64	32	32
Imagenet weights	Yes	Yes	Yes	Yes	Yes	Yes
Normalization	Yes	Yes	Yes	Yes	Yes	Yes
Optimizer	Adam	Adam	Adam	Adam	Adam	Adam
Learning rate (LR)	0.0001	0.0001	0.0001	0.0001	0.0001	0.0001
LR scheduling	None	None	None	None	None	None

References

[1] Ronneberger O, Fischer P, Brox T. U-Net: Convolutional Networks for Biomedical Image Segmentation. In: Medical Image Computing and Computer-Assisted Intervention (MICCAI). vol. 9351 of LNCS. Springer; 2015. p. 234–241. Available from: `http://lmb.informatik.uni-freiburg.de/Publications/2015/RFB15a`.

[2] Kingma DP, Ba J. Adam: A Method for Stochastic Optimization. In: Bengio Y, LeCun Y, editors. 3rd International Conference on Learning Representations, ICLR 2015, San Diego, CA, USA, May 7-9, 2015, Conference Track Proceedings; 2015.Available from: `http://arxiv.org/abs/1412.6980`.
