# Supplementary material for: A data science approach for multi-sensor marine observatory data monitoring cold water corals (Paragorgia arborea) in two campaigns
Source: PLoS One. 2023 Jul 19;18(7):e0282723. doi: 10.1371/journal.pone.0282723 (PMC10355400; doi:10.1371/journal.pone.0282723)
Supplement: S2 Text — A description of the method used for generating image patches to be used as classification model input. (PDF) [file pone.0282723.s006.pdf]

S2 Text: Patch generation for polyp activity classification

Our method for patch generation can be applied using manually annotated masks or masks generated e.g. by a CNN. As the masks generated by the segmentation models are smaller than the original images, they are upscaled before patch generation. For a given coral c, patches $D^i_{j=0,\ldots,n_i}$ from an image I_i are generated as follows: An image patch is defined as

$$D^i_j = \{I_i(x, y) | x_0(D^i_j) \leq x < x_0(D^i_j) + 128, \quad (1)$$

$$y_0(D^i_j) \leq y < y_0(D^i_j) + 128\} \quad (2)$$

and can be written as $D^i_j = (I_i, x_0, y_0)$, where (x_0, y_0) define the top left corner of D^i_j in I_i. Each image patch has a corresponding mask patch $Q^i_j = (M_i, x_0, y_0)$, which is defined accordingly. Each patch D^i_j is assigned a weight $w^i_j \in [0, 1]$ according to the share of pixels labeled as c in the corresponding mask patch Q^i_j.

Positions for patch extraction are defined as follows: First, a rectangular patch extraction region B_i is defined in image I_i. B_i is defined as a box around the center of the bounding box of the region segmented as coral c $O_i = \{(x, y)|M_i(x, y) = L(c)\}$ such that all pixels in O_i lie within B_i. The edge lengths of B_i are multiples of 128. If B_i overlaps with an edge of I_i, it is shifted such that is completely located within I_i.

Prior to extracting the patches, the background in the image is masked in order to remove unnecessary information. The masked image I'_i is defined as follows:

$$I'_i(x, y) = \begin{cases} I_i(x, y), & (x, y) \in O_i \\ (0, 0, 0), & \text{else.} \end{cases} \quad (3)$$

The content of O_i in I'_i is subdivided into square patches $D^i_{j=0,\ldots,n_i}$ without an overlap of the extracted patches.
