# Supplementary material for: A data science approach for multi-sensor marine observatory data monitoring cold water corals (Paragorgia arborea) in two campaigns
Source: PLoS One. 2023 Jul 19;18(7):e0282723. doi: 10.1371/journal.pone.0282723 (PMC10355400; doi:10.1371/journal.pone.0282723)
Supplement: S3 Text — A more detailed description of how the blue coral was processed. (PDF) [file pone.0282723.s007.pdf]

S3 Text: Polyp activity estimation for the "blue" coral C_b

The coral C_b is only visible in images recorded from camera viewing angles θ_1 and θ_2 during Γ'_1 and Γ_2. In images recorded by the optical stereo camera sensor K_0 from viewing angle θ_1, the visible part of C_b is small, while K_1 recorded the entire coral C_b from this viewing angle. In addition, many images recorded during Γ_1 do not show C_b, but for these images, no information on the camera position is given, i.e the images would need to be viewed to see if they show coral C_b and thus cannot be automatically excluded from the analysis.

To reduce the risk of falsely segmenting C_b in images where in fact it is not present, we exclude all images for which less than 1000 C_b pixels were segmented before activity estimation. Images with more than 30000 C_b pixels segmented are also excluded as they are considered outliers. Sizes are computed based on the segmentation masks with a size of 551×688. In addition, we only analyze θ_2 images from stereo camera sensor K_0 and θ_1 and θ_2 images from stereo camera sensor K_1 with respect to C_b for period Γ_2. To differentiate between camera angles is not necessary for Γ'_1 as the smaller regions are already excluded using the lower threshold of 1000 pixels. For C_r, we use all available images captured before 7 April 2019 and do not apply any thresholds.
