# Supplementary material for: A data science approach for multi-sensor marine observatory data monitoring cold water corals (Paragorgia arborea) in two campaigns
Source: PLoS One. 2023 Jul 19;18(7):e0282723. doi: 10.1371/journal.pone.0282723 (PMC10355400; doi:10.1371/journal.pone.0282723)
Supplement: S4 Text — Jaccard scores, precision, recall, and F1 scores definition. (PDF) [file pone.0282723.s008.pdf]

S4 Text: Evaluation metrics and accuracy assessment

The segmentation models are evaluated using the Jaccard score J [1], which can be defined for a class l (i.e. for the binary case) as

$$J_l = \frac{TP_l}{TP_l + FP_l + FN_l}, \tag{1}$$

where TP_l, FP_l, and FN_l are true positive, false positive, and false negative classifications , here pixel classifications with respect to class l [2, 3].

For evaluation of the classification models, we use the F_1-score (also called F-measure), which is based on the precision (P) and recall (R) measures. Their definition with respect to a given class l are as follows [3, 4, 5, 6]:

$$P_l = \frac{TP_l}{TP_l + FP_l} \tag{2}$$

$$R_l = \frac{TP_l}{TP_l + FN_l}, \tag{3}$$

$$F_{1,l} = \frac{2P_l R_l}{P_l + R_l} = \frac{2TP_l}{2TP_l + FP_l + FN_l}, \tag{4}$$

To assess the overall performance of a segmentation or classification model, we use the macro-average of the performance measures introduced above. The macro-averaged performance measure is an unweighted average of the performance measures for each class. For a performance measure E, the macro average will be denoted as $\bar{E}$ and is defined as follows [3, 7, 8]:

$$\bar{E} = \frac{1}{N} \sum_{l=1}^{N} E_l, \tag{5}$$

where N is the number of classes available.

References

[1] Jaccard P. The Distribution of the Flora in the Alpine Zone. The New Phytologist. 1912;11(2):37–50.

[2] Taha AA, Hanbury A. Metrics for evaluating 3D medical image segmentation: analysis, selection, and tool. BMC Medical Imaging. 2015;15:29. doi:https://doi.org/10.1186/s12880-015-0068-x.

[3] Lipton ZC, Elkan C, Naryanaswamy B. Optimal Thresholding of Classifiers to Maximize F1 Measure. In: Calders T, Esposito F, Hüllermeier E, Meo R, editors. Machine Learning and Knowledge Discovery in Databases. Berlin, Heidelberg: Springer Berlin Heidelberg; 2014. p. 225–239.

[4] Fawcett T. Introduction to ROC analysis. Pattern Recognition Letters. 2006;27:861–874. doi:10.1016/j.patrec.2005.10.010.

[5] Sokolova M, Lapalme G. A systematic analysis of performance measures for classification tasks. Information Processing & Management. 2009;45:427–437. doi:10.1016/j.ipm.2009.03.002.

[6] Ferri C, Hernández-Orallo J, Modroiu R. An experimental comparison of performance measures for classification. Pattern Recognit Lett. 2009;30:27–38.

[7] Tsoumakas G, Katakis I, Vlahavas I. Random k-Labelsets for Multilabel Classification. IEEE Transactions on Knowledge and Data Engineering. 2011;23(7):1079–1089. doi:10.1109/TKDE.2010.164.

[8] Yang Y. An Evaluation of Statistical Approaches to Text Categorization. Inf Retr. 1999;1(1–2):69–90. doi:10.1023/A:1009982220290.
