# Supplementary material for: A data science approach for multi-sensor marine observatory data monitoring cold water corals (Paragorgia arborea) in two campaigns
Source: PLoS One. 2023 Jul 19;18(7):e0282723. doi: 10.1371/journal.pone.0282723 (PMC10355400; doi:10.1371/journal.pone.0282723)
Supplement: S5 Text — A description of parameter and feature selection scheme for the LSTM models. (PDF) [file pone.0282723.s009.pdf]

S5 Text: Hyperparameter and feature selection for LSTM

First, we tested several parameter combinations for LSTM. Second, we tested all combinations of the input features depth, temperature, and the three current components v_1, v_2, and v_3. Third, we tested all combinations of the best ten parameter configurations and the best ten feature combinations. The parameter and feature combination achieving the lowest mean absolute error (MAE) predicting $\hat{a}(t)$ for the test time period from 22 March 2018, 15:00 until 12 April 2018, 14:00 was used to generate the experiment results described in Section 4.5.

Our approach for polyp activity prediction using LSTM models (sliding window) is explained in Section 3.6. As a loss function, the mean absolute error is used. For a list of all optimized parameters see Table 1.
The LSTM model architecture finally used consists of two LSTM layers in a row, each layer having an eight-dimensional hidden state. The last layer is connected to a single neuron that outputs the predicted activity value.

Table 1. Selected parameters for the LSTM models.

Parameter	Value
Optimizer	Stochastic gradient descent (SGD) with momentum=0.9
Learning rate	0.01
Subseries length η	6
Training Epochs per model $h_{t'}$	100
Hidden state dimensions	8
LSTM layers	2
Batch size	32

For further information on the SGD optimizer and momentum, please refer to [1].

References

[1] Sutskever I, Martens J, Dahl G, Hinton G. On the importance of initialization and momentum in deep learning. In: Dasgupta S, McAllester D, editors. Proceedings of the 30th International Conference on Machine Learning. vol. 28 of Proceedings of Machine Learning Research. Atlanta, Georgia, USA: PMLR; 2013. p. 1139–1147. Available from: `https://proceedings.mlr.press/v28/sutskever13.html`.
