# Supplementary material for: A data science approach for multi-sensor marine observatory data monitoring cold water corals (Paragorgia arborea) in two campaigns
Source: PLoS One. 2023 Jul 19;18(7):e0282723. doi: 10.1371/journal.pone.0282723 (PMC10355400; doi:10.1371/journal.pone.0282723)
Supplement: S6 Text — Additional details of sensor data preprocessing, including a description how gaps in the sensor data time series are handled during LSTM training and prediction. (PDF) [file pone.0282723.s010.pdf]

S6 Text: Preprocessing of sensor data in LSTM training and prediction

The LSTM architecture we use has a fixed input vector size. Therefore, measurements for all input features have to be available for all time points in an input subseries S_t for predicting polyp activity $\hat{a}(t)$. If a feature is missing at time point t, the full input vector $s(t)$ is removed, leaving a gap in the time series of input features. Standardization of the input features in $s(t)$ is done after removing values for time points t for which any feature is missing.

If a gap in time series $s(t)$ is smaller than 8 consecutive hours, the sensor data from the η previous, not consecutive, time steps available are used for predicting the polyp activity $\hat{a}(t)$ instead. If the gap is larger than 8 hours, no activity $\hat{a}(t)$ is predicted. The next t to predict $\hat{a}(t)$ for is selected such that η input vectors that are either consecutive or divided by a gap not larger than 8 hours are available as LSTM input.
