# Supplementary material for: A data science approach for multi-sensor marine observatory data monitoring cold water corals (Paragorgia arborea) in two campaigns
Source: PLoS One. 2023 Jul 19;18(7):e0282723. doi: 10.1371/journal.pone.0282723 (PMC10355400; doi:10.1371/journal.pone.0282723)
Supplement: S7 Text — A description of the smoothing process applied to polyp activity time series before visualization. (PDF) [file pone.0282723.s011.pdf]

S7 Text: Data smoothing for visualization

The smoothed time series plots shown in this paper (Fig 7, Fig 10, Fig 1 - Fig 5 in S2 Fig, Fig 1 in S6 Text) are generated as follows: Missing values are interpolated using linear interpolation if data for up to five hours are missing. If the gap is larger, the mean value of the time series is inserted in the full gap. The interpolated time series is smoothed using a Gaussian filter. After smoothing, all values inserted in the interpolation step are removed.
