# Supplementary material for: A data science approach for multi-sensor marine observatory data monitoring cold water corals (Paragorgia arborea) in two campaigns
Source: PLoS One. 2023 Jul 19;18(7):e0282723. doi: 10.1371/journal.pone.0282723 (PMC10355400; doi:10.1371/journal.pone.0282723)
Supplement: S8 Text — LSTM prediction and evaluation results and correlations of LSTM input sensor data and polyp activity for the blue Paragorgia colony Cb. (PDF) [file pone.0282723.s012.pdf]

S8 Text: LSTM prediction results and correlations for the blue *Paragorgia*

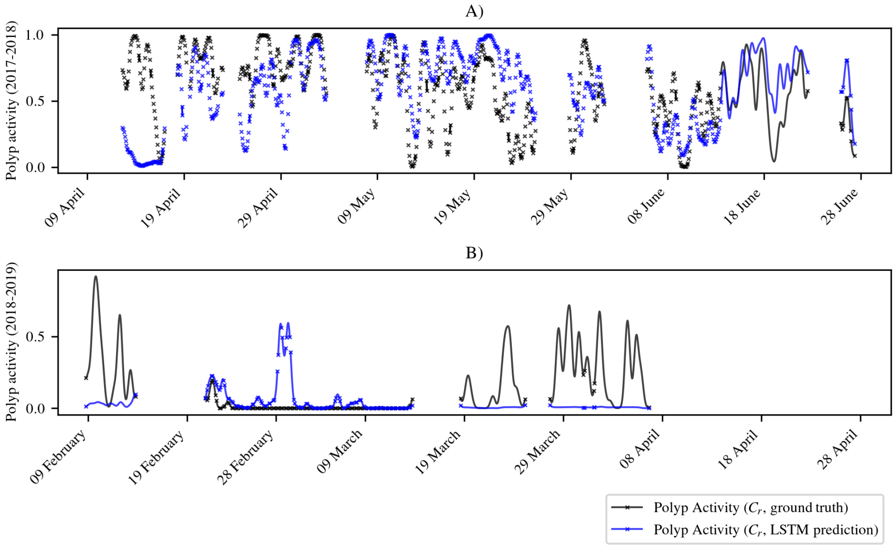

Fig 1. Smoothed result of polyp activity prediction for coral C_b, using our LSTM approach. Plots A) and B) show the results and ground truth for datasets $\mathcal{A}'_1$ and $\mathcal{A}'_2$, respectively. Smoothing was applied as described in S5 Text using a Gaussian filter with $\sigma = 5$. An "x" marker indicates the end of a series of consecutive hourly data points and thus a gap in the data.

$\mathcal{A}'_\tau$ refers to C_b polyp activity analogous to $\mathcal{A}_\tau$ for C_r polyp activity. The MAE for the "blue" colony C_b and $\mathcal{A}'_1$ is 0.344 ($r_S = 0.332,\ p = 1.2 \cdot 10^{-22}$). The MAE for C_b and $\mathcal{A}'_2$ is 0.166 ($r_S = 0.236,\ p = 8.7 \cdot 10^{-14}$). Correlations of "blue" coral activity with the feature time series can be found in Table 1. Correlations are calculated analogous to those for coral C_r (see Section 4.5). A plot of ground truth and predicted polyp activity of C_b can be found in Fig 1. For C_b activity prediction, the same set of input features was used as for C_r activity prediction.

Table 1. Spearman rank correlations and p-values.

	$\mathcal{A}'_1$ ($N = 826$)		$\mathcal{A}'_2$ ($N = 970$)	
Datatype	r_S	p	r_S	p
Depth	-0.046	0.184	-0.013	0.690
Temperature	-0.282	$1.4 \cdot 10^{-16}$	-0.046	0.148
Current velocity v_1	-0.170	$8.5 \cdot 10^{-7}$	-0.070	0.029
Current velocity v_2	0.190	$3.9 \cdot 10^{-8}$	0.158	$7.2 \cdot 10^{-7}$
Current velocity v_3	-0.277	$5.3 \cdot 10^{-16}$	0.016	0.624
Predicted activity	0.332	$1.2 \cdot 10^{-22}$	0.236	$8.7 \cdot 10^{-14}$

The table shows the Spearman rank correlations r_S between the ground truth polyp activity values for the blue *Paragorgia* C_b in the datasets $\mathcal{A}'_1$ and $\mathcal{A}'_2$ and the features used as input for polyp activity prediction using LSTM. Additionally, r_S between C_b ground truth polyp activity in $\mathcal{A}'_1$ and $\mathcal{A}'_2$ and the polyp activities predicted using our LSTM approach is shown. Accompanying p-values are shown for each correlation.
