# Supplementary material for: A data science approach for multi-sensor marine observatory data monitoring cold water corals (Paragorgia arborea) in two campaigns
Source: PLoS One. 2023 Jul 19;18(7):e0282723. doi: 10.1371/journal.pone.0282723 (PMC10355400; doi:10.1371/journal.pone.0282723)
Supplement: S1 Table — Jaccard scores, precision, recall, and F1 scores for test and validation of the segmentation models. (PDF) [file pone.0282723.s013.pdf]

S1 Table: Additional evaluation results for the segmentation models

Validation results and additional performance measures for the segmentation models.

Table 1. Jaccard scores per segmentation model and test or validation dataset.

Model	Measure	Class l	$\mathcal{I}_{1,\text{val}}$	$\mathcal{I}_{1,\text{test}}$	$\mathcal{I}_{1,\text{test}'}$	$\mathcal{I}_{2,\text{val}}$	$\mathcal{I}_{2,\text{test}}$
f_1	$\bar{J}$	-	(0.964)	0.947	0.840	0.638	0.672
f_2	$\bar{J}$	-	0.876	0.880	0.953	(0.937)	0.932
f_*	$\bar{J}$	-	(0.966)	0.925	0.961	(0.939)	0.939
f_1	J_l	$L(C_r)$	(0.968)	0.962	0.951	0.886	0.886
f_2	J_l	$L(C_r)$	0.954	0.949	0.956	(0.935)	0.941
f_*	J_l	$L(C_r)$	(0.966)	0.964	0.960	(0.935)	0.943
f_1	J_l	$L(C_b)$	(0.938)	0.896	0.612	0.072	0.175
f_2	J_l	$L(C_b)$	0.702	0.718	0.922	(0.893)	0.870
f_*	J_l	$L(C_b)$	(0.945)	0.828	0.939	(0.900)	0.888

Values in brackets refer to datasets used for optimizing the respective model. If a performance measure is specific to a class label l, l is given in the column "Class l" (see also Eq (1) in the main document).

Table 2. Recall, Precision, and F_1 scores per segmentation model and test or validation dataset.

Model	Measure	Class l	$\mathcal{I}_{1,\text{val}}$	$\mathcal{I}_{1,\text{test}}$	$\mathcal{I}_{1,\text{test}'}$	$\mathcal{I}_{2,\text{val}}$	$\mathcal{I}_{2,\text{test}}$
f_1	$\bar{P}$	-	(0.979)	0.973	0.967	0.937	0.908
f_2	$\bar{P}$	-	0.913	0.935	0.969	(0.964)	0.970
f_*	$\bar{P}$	-	(0.985)	0.979	0.978	(0.967)	0.974
f_1	P_l	$L(C_r)$	(0.979)	0.974	0.957	0.900	0.900
f_2	P_l	$L(C_r)$	0.981	0.979	0.967	(0.959)	0.966
f_*	P_l	$L(C_r)$	(0.987)	0.983	0.972	(0.960)	0.970
f_1	P_l	$L(C_b)$	(0.962)	0.949	0.971	0.932	0.845
f_2	P_l	$L(C_b)$	0.772	0.840	0.945	(0.940)	0.952
f_*	P_l	$L(C_b)$	(0.977)	0.963	0.969	(0.948)	0.960
f_1	$\bar{R}$	-	(0.985)	0.973	0.867	0.677	0.713
f_2	$\bar{R}$	-	0.947	0.929	0.982	(0.970)	0.958
f_*	$\bar{R}$	-	(0.980)	0.943	0.982	(0.970)	0.962
f_1	R_l	$L(C_r)$	(0.989)	0.987	0.994	0.983	0.983
f_2	R_l	$L(C_r)$	0.971	0.969	0.988	(0.974)	0.974
f_*	R_l	$L(C_r)$	(0.979)	0.980	0.988	(0.973)	0.972
f_1	R_l	$L(C_b)$	(0.974)	0.941	0.623	0.073	0.181
f_2	R_l	$L(C_b)$	0.886	0.831	0.974	(0.947)	0.909
f_*	R_l	$L(C_b)$	(0.967)	0.854	0.968	(0.947)	0.922
f_1	$\bar{F}_1$	-	(0.982)	0.973	0.904	0.684	0.738
f_2	$\bar{F}_1$	-	0.929	0.932	0.976	(0.967)	0.964
f_*	$\bar{F}_1$	-	(0.983)	0.960	0.980	(0.968)	0.968
f_1	$F_{1,l}$	$L(C_r)$	(0.984)	0.980	0.975	0.940	0.940
f_2	$F_{1,l}$	$L(C_r)$	0.976	0.974	0.978	(0.966)	0.970
f_*	$F_{1,l}$	$L(C_r)$	(0.983)	0.982	0.980	(0.966)	0.971
f_1	$F_{1,l}$	$L(C_b)$	(0.968)	0.945	0.759	0.135	0.298
f_2	$F_{1,l}$	$L(C_b)$	0.825	0.836	0.959	(0.944)	0.930
f_*	$F_{1,l}$	$L(C_b)$	(0.972)	0.906	0.968	(0.947)	0.941

Values in brackets refer to validation datasets used for optimizing the respective model. If the applied performance measure is specific to one class label l, l is given in the column "Class l" (see also Eq (1) in the main document).
