# Supplementary material for: A data science approach for multi-sensor marine observatory data monitoring cold water corals (Paragorgia arborea) in two campaigns
Source: PLoS One. 2023 Jul 19;18(7):e0282723. doi: 10.1371/journal.pone.0282723 (PMC10355400; doi:10.1371/journal.pone.0282723)
Supplement: S2 Table — (PDF) [file pone.0282723.s014.pdf]

S2 Table: Test and validation macro-averaged F_1 scores ($\bar{F_1}$) of the classification models.

Model	Coral	$\mathcal{D}^c_{1,\mathrm{val}}$	$\mathcal{D}^c_{1,\mathrm{test}}$	$\mathcal{D}^c_{1,\mathrm{test}'}$	$\mathcal{D}^c_{2,\mathrm{val}}$	$\mathcal{D}^c_{2,\mathrm{test}}$
$g_{1,r}$	C_r	(0.973)	0.963	0.979	0.962	0.936
$g_{1,b}$	C_b	(1.000)	0.957	0.944	0.888	0.895
$g_{2,r}$	C_r	0.972	0.952	0.990	(0.983)	0.963
$g_{2,b}$	C_b	0.948	0.905	0.923	(0.957)	0.952
$g_{*,r}$	C_r	(0.975)	0.968	0.990	(0.982)	0.960
$g_{*,b}$	C_b	(0.986)	0.957	0.929	(0.973)	0.951

Values in brackets indicate that the dataset was used for optimizing the tested model. Note that cross-time period validation sets can be seen as additional test sets as these sets were not used for optimizing the respective models.
