# Supplementary material for: A data science approach for multi-sensor marine observatory data monitoring cold water corals (Paragorgia arborea) in two campaigns
Source: PLoS One. 2023 Jul 19;18(7):e0282723. doi: 10.1371/journal.pone.0282723 (PMC10355400; doi:10.1371/journal.pone.0282723)
Supplement: Attachment — Submitted filename: Response to reviews.pdf [file pone.0282723.s019.pdf]

Response to reviews

First of all, we have to thank both reviewers for their thorough review and their helpful detailed comments. We are grateful for the chance to improve our manuscript with your help.

Reviewer #1:

This is a detailed and interesting analysis that tackles the tricky problem of model generalization and accuracy under changing observation conditions (a common issue with long-term underwater image sets). I applaud the authors on their detailed explanation of the methods and application of models trained on one time period to data collected in another in order to demonstrate the general applicability of this approach.

Response: The authors thank the reviewer for this comment.

My main issue with this study is that the model was trained over multiple time periods and conditions, but only on two individual organisms (one red and one blue Paragorgia), one of which it did not perform as well on for a number of reasons related to the quality and quantity of the data. This makes it somewhat harder to argue that this is a "generalized" model. The authors should be upfront about the fact that the model was trained on many varied observations of only two individuals, and the manuscript might benefit from framing this as a case study or proof-of-concept (especially given that the apparent correlation between polyp activity and conditions like temperature and current highlighted in the Discussion is based only on observations from one individual.)

I do not think that the limited number of individuals invalidates the approach or the value of this specific study, but it is an important caveat that deserves more acknowledgement early in the manuscript, ideally in the Introduction around lines 75 or 83 or at the beginning of the Materials/Methods (to the authors' credit, it is made clear in the abstract).

Response: Of course, we fully agree to every word here. We have added or re-phrased text accordingly in the introduction and in the discussion, as the reviewer proposes. In addition, we have changed the title of the manuscript a little to to emphasize the methodological part of the work.

Abstract:

"While some works of been published…" should be "have been published".

Response: This has been corrected. Thank you.

In general, I think the abstract could be made much shorter. There are sections that are redundant or too conversational for a concise summary of the study at hand.

Response: We have shortened the abstract by 3-4 lines and hope that the new version is better now.

Introduction:

The introduction suggests that the value of this type of study/methodology is to obtain more information on the ecology and behavior of Paragorgia via monitoring, but it is unclear how the data from a FUO is likely to contribute to this. It would be good to mention that this study is aimed at analyzing polyp activity specifically (and why we care about that) early in the introduction (closer to line 17 than line 83, where the subsection on this begins). The introduction fails to clearly establish a link between the need for better monitoring of Paragorgia and similar cold water corals and the way the data analyzed in this paper actually contributes to that. If the goal is to observe specific individuals and their feeding behavior (or other metrics like growth/survival/spawning/etc.) over time from a fixed location, this should be made clear (as well as how the proposed image analysis facilitates this).

Response: Thank you for your comment. We agree that a specific motivation for monitoring of polyp activity was missing. We have added a few lines to bridge this gap in the first paragraph of the introduction.

The two subsections beginning at lines 50 and 83 are a confusing mix of details on prior models and approaches and more general information about the data being collected and the behavior and ecology of the target species. Many of the modeling details in the second subsection feel like they belong in the first, which suggests that maybe the biological subsection should be presented before the machine learning/monitoring subsection with details on analyzing the data. In general, I believe the introduction would benefit from reorganization so that 1) the biological relevance of collecting/analyzing this data is established earlier, and 2) the ecology of the target species is described separately from the technical details on past and present modeling efforts.

Response: Thank you for this comment. We now have re-organized the introduction and put all biological background and motivation in the beginning of the Introduction.

Line 4: Can you offer a more specific depth range for this species (or, barring that, a definition of the depths referred to as "deep sea")?

Response: We have added this information now (> 200m).

Lines 43-45 It is unclear why having multi-sensor data (e.g., temperature time series and HD images) precludes direct processing. I am not quite sure what the authors are trying to say here. There are a number of reasons why multiple data types might be challenging to process or combine, as well as other challenges to processing the sheer volume of observations obtained over a long deployment, but that is not quite what this sentence seems to be saying. This is an important statement to get right, since it establishes the necessity and value of the rest of this study's analysis/methodology. (See my comment on Lines 508-511.)

Response: The reviewer is right, that we could have made this point more clearly. The main challenge here is that all different kinds of data have individual temporal resolutions,

dimensions / scales, variances, noise levels, missing values, etc. We have re-phrased this sentence now and provide a more detailed explanation.

Materials:

Line 116: If the specific title "Materials" is not required for this section, a more descriptive title like "Data" or "Data Collection" could be more appropriate.

<u>Response:</u> This is a nice idea. If the editor does not object, we change the title of this subsection to Data Collection. In the Instruction to authors / Manuscript organization it is stated that this is allowed/possible.

Methods:

Pay attention to tense throughout this section. Different subsections are written in different verb tenses, which is somewhat confusing, given that they all appear to describe different steps of the same analysis.

<u>Response:</u> The reviewer is right, and we have checked the text again and changed the tenses accordingly.

Lines 186 and 220: Given the number of images in the overall dataset, these seem like small numbers of images for training the model (although the level of detailed annotation required for each image may justify this). By my count, 205 total images were hand-classified for training, validation, and testing between the two time interval groups and the additional special interval Γ'1. Is this correct? If yes, it could be helpful to reiterate these total image counts on lines 218-222 when discussing manual labeling.

<u>Response:</u> The number of images is high enough. For the classification task, image patches are considered, and the number of patches is much higher than the number of images.in case of the red coral in the front, 100-150 patches were extracted from each training image. For the blue coral in the back, it was approx. 20 patches per image in the training set.
For the U-net segmentation, only a small number of training images is required. We have added a small text in 218-222 to relate to the original numbers in the beginning of the Methods section.

Results:

There are a number of subsections here where methods are reported alongside results. Methods should always be detailed in the Methods section and only restated here if necessary for understanding the results being reported. If these methods are being reported here because they apply to post-hoc analyses based on earlier results and were not part of the original study design, that should be made clear so that the reader can follow the rational for these additional analyses. I detail a couple of these instances below.

<u>Response:</u> We have re-edited this section accordingly, especially the parts the reviewer mentions in the following.

Lines 350-359: This section belongs in the Methods, not the Results. It is unclear to me why this entire subsection (beginning line 342) is in the Results and not the Methods, apart from the results discussed on lines 361-364.
Response: We have moved this part into the Methods section.

Lines 366-370, 407-411 and 427-429: Again, these details should already have been covered in the Methods, although briefly restating them here to aid the reader in understanding the reported values may be okay.

Response: We have integrated these text passages into the Methods sections now.

Discussion:

Lines 432-436: While restating other details of the analysis, this would be a good place to include that observations were made of two individual colonies.

Response: This has been done now and we have added text accordingly.

Line 451: These limitations that lead to lower accuracy for the blue Paragorgia are important to emphasize. Since only two individuals were analyzed here, it is likely that similar issues to those encountered with the blue Paragorgia would be relatively common should this model be applied to a larger dataset of different individuals in different conditions. While discussing how generalized the model is (regarding different time points and conditions), it is important to also acknowledge the limitations of this effort (regarding distinct individuals and poorer performance with distance, lighting, certain angles, etc.).

Response: We totally agree to the reviewer, which is why we have discussed the lower performance of the blue coral here in detail and say that these limitations should be considered. We have added an extra sentence to make this clearer.

Lines 465-472: These details should ideally be reported in the Results, rather than the Discussion.

Response: We have moved this part into the Results now.

Conclusion:

Lines 508-511: I think this may be what lines 43-45 were trying to say.

Response: Yes, and we have re-edited these lines now (see above).

Lines 524: While the results here are consistent with these previous observations, I do not believe they can be said to "confirm" such hypothesized relationships, given that the data comes from only one individual.

Response: We agree. This has been re-edited now.

General Observations:

There is some inconsistency between the references to figure numbers in the text (e.g., Figure 1 vs. Fig. 1).

Response: *We have checked this now.*

In general, this manuscript would benefit from proof-reading/copy editing to correct multiple small typos and cases of incorrect grammar. I have listed some lines on which I noted issues here, although the list is likely not comprehensive:
Lines 36, 38, 43, 71, 81, 85, 135, 179, 182, 183, 189, 218, 233, 241, 257, 290, 339, 477, 528, 532

Response: The entire manuscript including the lines mentioned by the reviewer (thank you very much for this!).

Reviewer #2:

This article answers a fundamental issue faced by scientists using images to monitor ecological phenomena: the bottleneck formed by data extraction from images. It presents a sophisticated method to automatically measure the level of soft corals polyps activity and then tests a modelling method to predict this activity as a function of the other parameters measured continuously by the observatory they are using. The manuscript is well written and draws appropriate conclusions. I see no issue with the method or the results and their interpretation. I, therefore, think it is worthy of publication.

Response: We thank the reviewer for this positive evaluation and recognition of our work.

However, I found this article hard to read given the complexity of the method and I found myself frequently coming back to the method section while reading the results to check the meaning of the many symbols, equations and formulas. This does not, by any mean, compromises the validity of the study and I acknowledge it is the way computer-science methods are reported, but I think additional comments in the method and results section highlighting the meaning of the different terms of jargon, parameters and metrics in a more plain language would make for smoother reading for an audience interested in this new way of monitoring coral physiology but less versed in computer sciences than the authors are. After all, the authors felt the need to define terms like semantic segmentation and fine-tuning so, they could expend on this didactic endeavor.

Response: We thank the reviewer for this advice, and we have re-edited some parts accordingly.

The discussion is short and makes minimal statements on the ecological implications of the potential application of this method in further ecological studies. This is not an issue but as a biologist who would potentially want to use this method to investigate corals activity in

relation to their environment, I wonder how much work will be needed to re-create this method? Could you expend on how accessible this method would be?

Response: This depends of course on the meaning of “re-create this method”. If a similar set up with similar hardware is applied to record CWC images over time with similar (or even better quality), there is no limitation at all. You start with collecting the data, annotate the training data and start the machine learning for both tasks (segmentation and polyp activity classification) using any appropriate publicly available deep learning framework. We have extended the discussion with some related points in this regard (see the middle and end of the Discussion). In case the images are of very similar quality and showing *P. arborea* it may not be necessary to train any new network but use the trained ones used in this study. If for instance, we would be provided with a third period of images from the same observatory, showing the same corals we would use the “old” trained networks first before collecting new training data.

Besides, this method seems heavily tailored to this specific case study given the method complexity, could all the steps be transferred to other cases without additional investigation and tuning?

Response: This depends on the meaning of the terms “other cases” and “tuning”. If you try to apply the methods (i.e. the segmentation and polyp activity classification networks) to new images recorded at different sites, showing other kinds of corals (like *L. pertusa*) it will not work. If you apply it to another *P. arborea* colony , recorded at a different location but with a similar camara setting (distance, illumination) we would expect a good performance, as our results are indicating. And if you use the whole method described in this paper again, i.e. including collecting training data and re-train the networks , we would be confident that the method would work for other corals too, if images are of sufficient quality.
We have re-phrased some parts in the discussion to make this clearer now.

Overall, opening-up to a less specialized audience by rephrasing or extending some passafes would help the diffusion of this method and better advocate its potential to the community that would use these results. I have made some suggestions towards better clarity of the text as well as some very minor typos in the text that should be corrected before publication.

Response: All technical / algorithmic parts have been checked and each time when it seemed appropriate, we have added text, explaining the method without avoiding technical 7 algorithmic jargon.

14: on those

Response: Has now been corrected. Thank you.

14-16: awkward phrasing

Response: Has been re-phrased. Thank you.

36: “data from both the cameras and other sensors provide a valuable… “

Response: Has been re-phrased. Thank you.

38 approaches in data

Response: Has now been corrected. Thank you.

38: 5 data in 1 sentence. “the heterogeneity in the in the data calls for special approach…”

Response: Has been re-phrased. Thank you.

61: “the recorded time series spans less than a year”

Response: Has been re-phrased. Thank you.

67: break sentence in 2.

Response: Has been done and re-phrased. Thank you.

78: the monitoring of a larger area

Response: Has now been corrected. Thank you.

81: method to extract

Response: Has now been corrected. Thank you.

85: the question whether xxxx or not

Response: Has been re-phrased. Thank you.

105: If you are mentioning it, Can you succinctly define single features vectors? Their time lag?

Response: Has been added. Thank you.

110: target output = response variable that you are trying to predict? This is the pivotal paragraph where short clear sentences would make sure readers get the objectives.

Response: Has been re-phrased. Thank you.

186: not sure how the numbering works here as there are 13 lines between 186 and 187… Anyway, in that paragraph, it is stated 100 images were selected. Is that a random pick within all the available images and a further random split? Or was there an attempt to homogenize the representativity of the different states of the colonies in each one of the 3 sets?

Response: It is a random selection of 60+20+20 images. The part has been re-phrased. Thank you.

Also, was it another 100 images selected for each interval?

Response: Yes, the same was done for the other interval too in order to compare the generalization performance of the computer vision from interval 1 to interval 2 and vice versa.

201: is there a reason for these specific numbers? – also, do you have an estimate of how much processing time was saved?

For the downscaled images, the processing time for the U-Net segmentation was on average 45 images per sec. This was approx. 10-times faster then for the original images. But this factor also depends on other factors like GPU hardware. The size 551 × 688 is a result of subsampling by factor 4 in both dimensions.

218: numbering stops again. I feel it would help some of the audience unfamiliar with mathematical annotations to lay down the meaning of the equations in plain text to ensure the pivotal principles used in the study are clear.

Response: More text has been added accordingly to make this easier to understand.

Where does the 20% come from?

Response: This threshold is based on the visual experience in annotation. If more than 20% of the polyps appear extended the confidence in the annotation was sufficient, i.e. the user was able to reproduce the annotation. This threshold is not very sensitive. We could set it a little lower but preferred to have a more “conservative” computational polyp activity assessment and avoid false positive classifications as much as possible. We have added an explanation to the text.

235: That is not an easy sentence to read.

Response: The reviewer is right and so we have re-phrased this sentence and split it into two.

250: is it all polyps retracted or there is no D patch where more than 20% of the polyps are extended?

Response: This sentence may be a little misleading. We have deleted it and replaced it with another one.

288: to compute??

Response: This has been re-edited now, thank you.

290: standardized to zero mean is zero and unit variance??? Does that need a rephrase?

Response: This has been re-edited / corrected now, thank you.

295: odd start of a paragraph. Can you say what the sliding window will be used for?

Response: The reviewer is perfectly right, this made no sense. Sorry, this must have been a copy-paste mistake! We have re-organized this paragraph.

482: rephrase sentence

Response: Done. Thank you. We just wanted to clarify, that the approach is not dependent on this observed kind of colony behavior, i.e. a synchronization of the polyp activity.

488: periods of inactivity?

Response: The reviewer is right. This has been re-edited.

520: maybe but what is too much? Can you give some concrete element of what that entails?

Response: This is difficult to determine. If for instance for each campaign and each coral colony training data must be collected by manual annotation, the costs for this kind of monitoring could be too high and outside a budget (especially in academic research!). So it would be preferable to reduce this effort and avoid as much manual labeling as possible. The manual collection of training data requires around 1-2 working days using a software like BIIGLE, including re-evaluations / reproducibility studies. We have added some text there to make our point clearer.

520: rephrase sentence. e.g.: "It also indicates that transferring this approach to other CWC species has good chances of success".

Response: This has been done. Thank you.

532: periods of camera failures

Response: This has been corrected. Thank you.
